# Association between sports participation and resilience in school-attending students: a cross-sectional study

**DOI:** 10.3389/fpsyg.2024.1365310

**Published:** 2024-04-25

**Authors:** Xinxin Sheng, Kaixin Liang, Kai Li, Xinli Chi, Huiying Fan

**Affiliations:** ^1^School of Physical Education, Changzhou University, Changzhou, China; ^2^School of Psychology, Shenzhen University, Shenzhen, Guangdong, China; ^3^School of Physical Education, Shanghai University of Sport, Shanghai, China; ^4^School of Physical Education, Shanghai Normal University, Shanghai, China

**Keywords:** sports participation, resilience, school-attending students, cross-sectional study, association

## Abstract

**Aim:**

This research sought to identify the association between sports participation and resilience in children and adolescents as a means to enhance mental health.

**Methods:**

A comprehensive survey was carried out, encompassing primary, middle, and high school students from chosen educational institutions. The analytical sample comprised 67,281 students of school age. Sports participation and resilience were evaluated using validated assessment tools, while relevant covariates, such as sex and school grade, were assessed through self-reported questionnaires. Generalized Linear Models were applied to ascertain the association between sports participation and resilience for the entire sample, and separately for subgroups divided by gender or school grade, after controlling for covariates.

**Results:**

Among the 67,281 school students, males constituted 51.9% of the sample. Approximately 47.1% of the entire sample reported no sports participation, and the average resilience score was 24.7. The regression model analysis revealed that, in the entire sample, increased in sports participation was linked to higher resilience scores (odds ratio [OR] for 1–3 times per month: 1.20, 95%CI: 1.16–1.24; OR for 1–2 times per week: 1.38, 95%CI: 1.33–1.43; OR for 3 times or more per week: 1.72, 95%CI: 1.65–1.79). Analyses stratified by gender and school grade indicated that sports participation was consistently associated with greater resilience.

**Conclusion:**

This study provides cross-sectional evidence supporting the positive association between sports participation and the resilience of children and adolescents, underscoring the potential of encouraging sports participation as a strategy for promoting mental health resilience. The findings presented herein should be subject to further confirmation or refutation in future research endeavors.

## 1 Introduction

Mental health issues among school-attending students are a subject of growing public concern due to their elevated prevalence. Despite children and adolescents having a lower likelihood of experiencing clinical mental disorders when compared to adults, recent studies have shown that mental health problems [e.g., depression (Wermelinger Ávila et al., 2018), mental well-being (Moore et al., 2020), affective disorders (Deng et al., 2023), etc.] are prevalent among school-attending students in a number of countries. Meanwhile, it is paramount not to overlook their mental well-being, given that almost 50% of psychiatric illnesses manifest prior to turning 18 (Solmi et al., 2022). Reinforcing the resilience of adolescents plays a crucial part in averting unfavorable mental health outcomes and promoting sustained well-being. Resilience refers to a person’s ability to successfully adjust to hardship in this context (She et al., 2020). Resilience is needed to cope with a wide range of adversities, from everyday distress to major life events (Fletcher and Sarkar, 2013). Research has indicated that adolescents with higher resilience levels exhibit reduced chances of developing emotional and behavioral issues (Chen and Kuo, 2019; Xiao et al., 2020). For example, adolescents with higher levels of resilience typically have higher levels of subjective well-being, life satisfaction (Moreira et al., 2021) and less suicidal behaviors (Sánchez-Teruel and Robles-Bello, 2014). Therefore, it is imperative to foster factors that can enhance the resilience of school-attending students.

Individual resilience is affected by a multitude of factors, including as genetic, biological, psychological, cultural and social aspects (Iacoviello and Charney, 2014; Niitsu et al., 2019). Among these factors, physical activity, as a modifiable element, has demonstrated promising associations with the mental health outcomes of adolescents. A growing corpus of data substantiates the significance of physical activity in fortifying resilience and, consequently, its positive impact on mental health (Ho et al., 2015; Belcher et al., 2021; Zhang et al., 2022). For instance, a study involving Hong Kong Chinese adolescents revealed a strong link found between physical activity levels and adolescents’ resilience. Furthermore, resilience was found to mediate the connection among mental health and physical activity (Ho et al., 2015). The findings of a meta-analysis supported the significant beneficial impacts of treatments including physical activity related to schooling on resilience (Andermo et al., 2020). Researchers have postulated that physical activity may enhance resilience by strengthening specific brain regions and neural circuits, as indicated by existing neuroimaging studies (Belcher et al., 2021). Consequently, physical activity has been established as advantageous for enhancing youngsters’ and adolescents’ mental health, contributing to the development of their resilience and other positive components that collectively constitute overall well-being.

Notably, prior research has demonstrated that distinct forms of physical activity may exhibit varying associations with mental health outcomes (Fong Yan et al., 2017; Koch et al., 2020). Sports participation, as a subcategory of physical activity, typically involves high physical exertion, competitive intentions, and well-defined rules (Koch et al., 2020). In contrast to physical activity in a broad context, sports participation often includes more significant social interaction and social support, potentially leading to more pronounced connections with mental health outcomes (Easterlin et al., 2019; Guddal et al., 2019; Chen et al., 2021). For example, an inquiry into adolescents with hearing impairments uncovered a stronger link between sports participation and a decreased likelihood of adverse psychosocial consequences (e.g., depression and anxiety) in comparison to participation in general physical activities (DeLuca and Rupp, 2022). These emerging findings emphasize the importance of understanding the connection between resilience and sports participation in children and adolescents, as well as more general physical activities.

Nevertheless, limited knowledge exists with respect to the relationship between resilience and sports participation. Considering that sports participation represents a form of physical activity aligned with the school context and can be easily implemented within groups of students, it becomes imperative to clarify the connection between adolescents’ in sports participation and their ability to cope with pressure. Furthermore, research has shown variations in levels of sports participation (Heradstveit et al., 2020) and resilience concerning age/grade and gender (Toftegaard-Støckel et al., 2011; She et al., 2020). For example, boys’ sport participation is typically higher than girls’, and sport participation decreases with grade level (Denham, 2014). Resilience is higher among girls than boys in primary schools, but this difference decreases with grade (Sun and Stewart, 2012). Although understudied, the association between sport participation and resilience may also vary by gender and grade stage. The association between sport participation and resilience may also be affected by gender and grade, making it necessary to study school-attending students of gender and grade.

Although understudied, the association between in sports participation and resilience may also potentially differ based on gender and developmental stages.

In light of the aforementioned background, with the help of a large sample of Chinese adolescents, this research intended to examine the association between sports participation and resilience. Our hypothesis posited that increased sports participation would be linked to improved resilience. Additionally, we explored whether the association between resilience and sports participation fluctuates based on school grade and gender. The findings would bear significance in the design of physical activity-based intervention programs aimed at bolstering the students’ resilience in China.

## 2 Techniques

### 2.1 Procedure and participants

In March 2021, an extensive survey was carried out in Shenzhen, one of China’s most developed cities. The study focused on students enrolled at nearby elementary and secondary public schools. The questionnaire employed an approach to sampling in two stages, involving the selection of specific survey areas and the identification of particular schools within these chosen areas. The study included students from primary, middle, and high school levels among the chosen schools.

This research encompassed students across primary, middle, and high school levels within specific educational institutions. The survey was aimed at adolescents aged 10 years and above, starting from the 5th grade. This age group was chosen due to their well-developed cognitive abilities, which ensured the smooth conduct of the survey. It is worth noting that students in the 8th and 11th grades were deeply engrossed in intensive preparations for senior high school and college entrance examination, leading to tightly packed schedules. Consequently, their participation in this extensive survey was limited.

Prior to the commencement of this investigation, comprehensive information was meticulously provided to all participants as well as their legal guardians, outlining the precise objective and substance of the questionnaire. It was emphasized that all participation was optional, and the information gathered would be used for research purposes only to rigorous anonymization protocols. To facilitate the survey process, dedicated homeroom teachers guided willing students in completing the online questionnaire independently within the confines of a computer room, with the average duration of participation spanning approximately 20 min. The survey was meticulously conducted through the utilization of a prominent Chinese survey platform, known as “Wenjuanxing.”

In alignment with ethical standards and compliance with pertinent regulations, the guardian consent of all the participants had been obtained before the survey began. After the guardian’s consent, the staff distributed the e-questionnaire to the respondents through an online format, an electronic informed consent form was prominently displayed to participants prior to their access to the survey page. Only those who willingly provided consent by clicking the “Agree” button were permitted to proceed to the subsequent answering phase. Electronic consent was obtained from all participants on the platform online. It is imperative to note that our questionnaire was painstakingly designed to fully conform to all applicable regulatory frameworks and had received official clearance from Shenzhen University’s Research Committee (Approval Number: 2020005). The comprehensive response dataset ultimately consisted of inputs from total of 78,428 students. Following the initial phase of data collection, a thorough data cleaning procedure was undertaken. This process entailed the exclusion of participant data with recorded response times falling below the minimum threshold of 300 s, resulting in the retention of dataset comprising 67,281 samples.

### 2.2 Measurements

#### 2.2.1 Sport Participation

The frequency of sports participation was ascertained through a singular survey item, wherein respondents were inquired: “To what extent have you engaged in sports teams or sports clubs over the course of the past year?” Participants were given the prerogative to select from a range of specified response choices, including “Never,” “1–3 times per month,” “1–2 times per week,” or 3 or more times per week” (She et al., 2020).

#### 2.2.2 Resilience

Resilience was evaluated using the 10-item Connor-Davidson Resilience Scale (CD-RISC-10). The scale comprises 10 items with 5 response options (0 = never, 4 = almost always). Greater resilience was indicated by a higher overall score. The CD-RISC-10 is a well-developed instrument for assessing resilience that has been validated in adolescent populations (Wollny and Jacobs, 2023; López-Fernández et al., 2024), and there is also evidence demonstrating the validation in Chinese adolescents (Chen et al., 2022).

#### 2.2.3 Controlling variables

Additional information was collected via self-administered questionnaires encompassing various demographic and background factors. These factors included gender (categorized as boy or girl), school grade (categorized as primary, middle, or high school), self-reported height and weight (in centimeters and kilograms), sibling status (categorized as whether an only kid or not), family structure (categorized as having both parents or a single parent), parental education level (categorized as middle school or below, high school or its equivalent, bachelor’s degree or higher, master’s degree or above, or not sure), ethnicity (categorized as Han or belonging to a minority group), family socioeconomic status (assessed utilizing the MacArthur Scale of Subjective Social Status, modified, which employs a scale ranging from 0 to 10) (Cundiff et al., 2013), geographic district (with a total of nine districts), and school attended (with a total of 135 schools).

Furthermore, age- and gender-specific weight status, as determined by the body mass index (BMI), was calculated using norm reference data for China (Zhu et al., 2019). Participants were stratified into one of three categories: normal weight, overweight, or obese.

### 2.3 Statistical analysis

The finally analytical sample was 67,281. All statistical analyses were carried out with STATA BE 17.0 (College Station, Texas, USA). The sample characteristics were reported using descriptive statistics. A three-level mixed multilevel effect model (level 3: district; level 2: school; level 1: individual; such layers were based on sampling strategy) was used to assess the associations of sports participation with resilience after controlling for all the covariates mentioned above. To achieve the analysis, *meglm* was used, ‘never participate in sports’ and ‘the lowest level in the measure of resilience’ was treated as reference group in the regression model analysis, respectively. Analysis for the whole sample and sample by sex and school grade were performed, separately. Results are presented as a 95% confidence interval (CI) and odds ratio (OR). For statistical significance, a *p*-value of less than 0.05 was used.

## 3 Results

This study encompassed 67,281 children and adolescents, with an average age of 13.0 ± 1.8 years (see Table 1). From the participants in the research, 48.1% were female, and 96.6% belonged to the Han ethnic group. The distribution of students was as follows: 41.6% in primary school, 40.3% in middle school, and 18.1% in high school. Notably, the overweight percentage was 13.5, and 18.4% were classified as obese, while the majority of adolescents, 68.1%, had a normal weight. 25.8% of the attendees reported having no siblings, and 93.4% of the adolescents had a two-parent family structure. The average subjective family socioeconomic status score was 5.0 ± 1.7. In terms of paternal education levels, 21.7% had completed education up to high school or below, 27.0% had attained education equivalent to high school, 38.7% held a bachelor’s degree or its equivalent, and 4.2% had achieved a master’s degree or higher. For maternal education levels, 26.2% had completed education up to junior high school or below, 27.8% had attained education equivalent to high school, 35.6% held a bachelor’s degree or its equivalent, and 2.4% had achieved a master’s degree or higher. With regard to sports participation, 22.4% of adolescents participated in sports 1–3 times per month, 17.5% took part once or twice a week, and 13.0% took part three or more times per week. Unfortunately, 47.1% of the adolescents reported never participating in any sports. The average resilience score was 24.7 ± 8.4.

**Table 1:** Sample characteristics of study participants in this study.

**Categorical variables**	***n***	**%**
Sex		
Boy	34,909	51.9
Girl	32,372	48.1
Grade		
Primary school	27,954	41.6
Middle school	27,124	40.3
High school	12,203	18.1
Weight status		
Normal	45,817	68.1
Overweight	9,051	13.5
Obese	12,413	18.4
Siblings		
Only child	17,354	25.8
Non-only child	49,927	74.2
Family structure		
Full	62,836	93.4
Other	4,445	6.6
Paternal education		
Junior high school or below	14,619	21.7
High school or equivalent	18,159	27.0
Bachelor or equivalent	26,030	38.7
Master or above	2,796	4.2
Unclear	5,677	8.4
Maternal education		
Junior high school or below	17,617	26.2
High school or equivalent	18,706	27.8
Bachelor or equivalent	23,922	35.6
Master or above	1,635	2.4
Unclear	5,401	8.0
Ethnicity		
Han	65,027	96.6
Minority	2,254	3.4
Sports participation		
Never	31,721	47.1
1–3 times per month	15,050	22.4
1–2 times per week	11,789	17.5
3 or more times per week	8,721	13.0

**Continuous variables**	**Mean**	**SD**
Age (years)	13.0	1.8
Subjective family socioeconomic status	5.0	1.7
Resilience	24.7	8.4

SD, standard deviation.

Table 2 summarizes in the whole sample, there is a correlation between sports participation and resilience and by sex. After controlling for covariates, the study found that greater resilience was positively associated with increased sports participation frequency. Specifically, children and adolescents who participated in sports 1–3 times per month were more likely to do so than those who did not (odds ratio [OR] = 1.20, 95% confidence interval [CI] = 1.16–1.24, *p* < 0.001), 1–2 times per week (OR = 1.38, 95% CI = 1.33–1.43, *p* < 0.001), and 3 or more times per week (OR = 1.72, 95% CI = 1.65–1.79, *p* < 0.001) were more likely to report greater resilience. In boys, sports participation and resilience were also shown to be positively correlated. Boys who participated in sports one to three times per month outnumbered those who did not participate in sports (OR = 1.12, 95% CI = 1.07–1.18, *p* < 0.001), 1–2 times per week (OR = 1.31, 95% CI = 1.25–1.38, *p* < 0.001), and 3 or more times per week (OR = 1.70, 95% CI = 1.60–1.80, *p* < 0.001) were more likely to report greater resilience. A similar association was found in girls. Girls who participated in sports 1–3 times per month (OR = 1.29, 95% CI = 1.23–1.35, *p* < 0.001), 1–2 times per week (OR = 1.46, 95% CI = 1.38–1.54, *p* < 0.001), and 3 or more times per week (OR = 1.75, 95% CI = 1.64–1.87, *p* < 0.001) were more likely to have greater resilience.

**Table 2:** Regression models results for association between sports participation and resilience in overall sample and by sex.

**Overall**					**Boys**					**Girls**				
	OR	95%CI		*p*		OR	95%CI		*p*		OR	95%CI		*p*
**Sports participation**					**Sports participation**					**Sports participation**				
1–3 times/month	1.20	1.16	1.24	<0.001	1–3 times/month	1.12	1.07	1.18	<0.001	1–3 times/month	1.29	1.23	1.35	<0.001
1–2 times/week	1.38	1.33	1.43	<0.001	1–2 times/week	1.31	1.25	1.38	<0.001	1–2 times/week	1.46	1.38	1.54	<0.001
3 times or more/week	1.72	1.65	1.79	<0.001	3 times or more/week	1.70	1.60	1.80	<0.001	3 times or more/week	1.75	1.64	1.87	<0.001
**Covariates**					**Covariates**					**Covariates**				
Sex	0.63	0.61	0.65	<0.001	Grade	1.06	0.99	1.13	0.113	Grade	1.05	0.97	1.14	0.210
Grade	1.07	1.01	1.13	0.026	Body mass index	0.91	0.89	0.93	<0.001	Body mass index	0.89	0.87	0.91	<0.001
Body mass index	0.90	0.89	0.92	<0.001	Family socioeconomic status	1.13	1.12	1.15	<0.001	Family socioeconomic status	1.14	1.12	1.15	<0.001
Family socioeconomic status	1.13	1.12	1.14	<0.001	Single child or not	0.84	0.81	0.87	<0.001	Single child or not	0.80	0.76	0.84	<0.001
Single child or not	0.82	0.80	0.85	<0.001	Father education level	1.00	0.97	1.02	0.757	Father education level	1.01	0.98	1.03	0.477
Father education level	1.00	0.99	1.02	0.782	Mother education level	1.00	0.98	1.02	0.934	Mother education level	0.97	0.95	0.99	0.012
Mother education level	0.99	0.97	1.00	0.079	Living with parents	0.84	0.78	0.91	<0.001	Living with parents	0.75	0.70	0.81	<0.001
Living with parents	0.80	0.75	0.84	<0.001	Race	1.03	0.93	1.15	0.525	Race	1.17	1.05	1.29	0.004
Race	1.10	1.02	1.18	0.015	Age	0.94	0.91	0.96	<0.001	Age	0.91	0.89	0.94	<0.001
Age	0.93	0.91	0.94	<0.001										

OR, odds ratio; CI, confidence interval. Reference group for sports participation: Never. Adjusted results were presented in the table.

Table 3 displays the associations between resilience and sports participation among different grade levels. Due to the differences in sports participation and resilience among adolescents of different ages (Van Tuyckom et al., 2010; Sun and Stewart, 2012), it is necessary to analyze adolescents of different ages separately. In primary school, children and adolescents who participated in sports one to three times a month (OR = 1.16, 95%CI = 1.10–1.23, *p* < 0.001), 1–2 times per week (OR = 1.33, 95%CI = 1.26–1.41, *p* < 0.001), and 3 or more times per week (OR = 1.77, 95%CI = 1.66–1.88, *p* < 0.001) had a higher likelihood of having greater resilience in contrast to those who did not participate in sports. Similar results were found in middle school students, with those who participated in sports 1–3 times per month (OR = 1.24, 95%CI = 1.17–1.31, *p* < 0.001), 1–2 times per week (OR = 1.38, 95%CI = 1.30–1.47, *p* < 0.001), and 3 or more times per week (OR = 1.63, 95%CI = 1.53–1.74, *p* < 0.001) being more resilient. In high school, adolescents who participated in sports 1–3 times per month (OR = 1.19, 95%CI = 1.11–1.29, *p* < 0.001), 1–2 times per week (OR = 1.47, 95%CI = 1.34–1.60, *p* < 0.001), and 3 or more times per week (OR = 1.74, 95%CI = 1.55–1.97, *p* < 0.001) also showed a positive association with greater resilience.

**Table 3:** Regression models results for association between sports participation and resilience in sample by grade.

**Primary school**					**Middle school**					**High school**				
	OR	95%CI		*p*		OR	95%CI		*p*		OR	95%CI		*p*
**Sports participation**					**Sports participation**					**Sports participation**				
1–3 times/month	1.16	1.10	1.23	<0.001	1–3 times/month	1.24	1.17	1.31	<0.001	1–3 times/month	1.19	1.11	1.29	<0.001
1–2 times/week	1.33	1.26	1.41	<0.001	1–2 times/week	1.38	1.30	1.47	<0.001	1–2 times/week	1.47	1.34	1.60	<0.001
3 times or more/week	1.77	1.66	1.88	<0.001	3 times or more/week	1.63	1.53	1.74	<0.001	3 times or more/week	1.74	1.55	1.97	<0.001
**Covariates**					**Covariates**					**Covariates**				
Sex	0.70	0.67	0.73	<0.001	Sex	0.58	0.56	0.61	<0.001	Sex	0.57	0.53	0.61	<0.001
Body mass index	0.89	0.86	0.91	<0.001	Body mass index	0.91	0.89	0.93	<0.001	Body mass index	0.95	0.91	1.00	0.046
Family socioeconomic status	1.13	1.12	1.15	<0.001	Family socioeconomic status	1.14	1.12	1.15	<0.001	Family socioeconomic status	1.12	1.09	1.14	<0.001
Single child or not	0.77	0.74	0.81	<0.001	Single child or not	0.83	0.79	0.87	<0.001	Single child or not	0.93	0.86	0.99	0.032
Father education level	0.99	0.97	1.02	0.483	Father education level	1.03	1.00	1.06	0.042	Father education level	0.98	0.94	1.02	0.301
Mother education level	0.99	0.97	1.02	0.492	Mother education level	0.98	0.95	1.00	0.086	Mother education level	1.00	0.96	1.04	0.927
Living with parents	0.73	0.67	0.80	<0.001	Living with parents	0.81	0.74	0.88	<0.001	Living with parents	0.87	0.78	0.98	0.019
Race	1.04	0.93	1.16	0.544	Race	1.20	1.07	1.35	0.002	Race	1.01	0.85	1.20	0.923
Age	0.92	0.90	0.94	<0.001	Age	0.91	0.89	0.94	<0.001	Age	0.97	0.93	1.02	0.225

OR, odds ratio; CI, confidence interval. Reference group for sports participation: never. Adjusted results were presented in the table.

## 4 Discussion

This study presents a significant association between sports participation and resilience among school-attending students in Shenzhen City, China, while controlling for a wide array of covariates, including factors like sex, grade, and BMI. The key findings can be summarized as follows: The main findings are that: (1) more sports participations were positively associated with higher scores of resilience; (2) the positive association between sports participation and scores of resilience were not varied across sexes and school grades; (3) positive cross-sectional association observed between sports participation and resilience. The following sections are the detailed analysis for research findings.

Some previous research have investigated the association between physical activity and resilience. For instance, data from a study on Hong Kong adolescents revealed a positive association between physical activity and resilience among adolescents, which aligns with our findings (Ho et al., 2015). Additionally, two studies involving adults also reported consistent results, indicating that greater resilience has been associated with greater levels of physical activity (Wermelinger Ávila et al., 2018; Zach et al., 2021). However, given the distinctions between sports participation and general physical activity, caution is necessary when drawing direct comparisons with prior research.

There are some hypothesized biological mechanisms underlying the association between sports participation and resilience, which are both multifactorial and complex. One of the possible mechanisms is that sports participation attenuates/optimizes neuroendocrine and physiological responses to physical and psychosocial stress (Silverman and Deuster, 2014). Regular sports participation leads to physiological “stress training” or “reinforcement.” This protective physiological profile appears to be associated with improved performance in challenging/stressful situations (Nabkasorn et al., 2006), increasing emotional stability and improving immune functioning (Sothmann et al., 1996). Another mechanism by sports participation can confer resilience through minimizing inflammation (Silverman and Deuster, 2014). For example, psychological stress and physical inactivity/low aerobic fitness have been associated with persistent systemic low inflammation and adverse effects on mental and physical health (Hamer, 2007). Pro-inflammatory cytokines can affect virtually every area of pathophysiology associated with depression, including neuroendocrine function, neurotransmitter metabolism, and neuroplasticity, and ultimately resilience (Thomson et al., 2009). Individuals who regularly engage in physical activity have lower concentrations of inflammatory biomarkers compared to inactive individuals (Plaisance and Grandjean, 2006; Hamer, 2007).

One of the pioneering investigations on the association between sports participation and resilience in children and adolescents, to the best of our knowledge, is represented by this study. Leveraging a substantial sample size, our research contributes to the growing reservoir of knowledge in this field. By concentrating on sports participation and its association with resilience, we have enhanced our understanding of the benefits of this specific form of physical activity. While previous literature has explored the link between physical activity or exercise and resilience, most of these investigations focused on the total amount of physical activity or exercise, rather than delving into the effects of specific physical activity modalities. Only a limited body of research, such as a recent intervention study that exclusively involved male adolescents, has addressed the promotion of resilience through sports participation (Vella et al., 2021). The findings from this intervention study corroborate our own, indicating that sports participation is indeed conducive to fostering resilience. Nonetheless, it is imperative to acknowledge the relative scarcity of studies on sports participation and resilience in children and adolescents. Thus, further research is warranted to advance our knowledge in this area. Considering the unique contexts and attributes of sports participation as a specific form of physical activity, future studies should encompass sports participation programs to determine the extent of its impact on promoting resilience among children and adolescents. Several underlying mechanisms concerning physical activity and exercise and their associations with resilience can shed light on the studied link between sports participation and resilience. Sports participation can be viewed as a specific form of physical activity or exercise, and understanding the mechanisms that connect physical activity and resilience can help elucidate why sports participation may contribute to resilience.

Firstly, it is well-established that adequate physical activity promotes physical fitness in children and adolescents (Shomaker et al., 2012; Williams et al., 2016). Elevated levels of physical fitness, coupled with regular physical activity (Biddle and Asare, 2011; Biddle et al., 2019), are known to be significant drivers of improved mental health, potentially enhancing resilience (Belcher et al., 2021). Secondly, from a biological and neurological perspective, participating in physical activity can bolster resilience by enhancing brain self-regulation functions. This enhancement can occur through the exertion of neuroplastic effects on neural circuits associated with self-regulation (Belcher et al., 2021). Nevertheless, these assumptions are primarily based on research regarding physical activity and resilience, which did not take into account the specific contexts in which physical activity occurs, such as sports participation. As sports participation often involves unique contexts (e.g., team sports are associated with social interactions), further research is essential to clarify the actual mechanisms connecting sports participation and resilience, particularly in light of contextual factors. Beyond uncovering the biological mechanisms linking resilience sports participation in children and adolescents, sociological mechanisms also warrant research attention.

This study revealed a positive association between sports participation and resilience in school-attending students, which was consistently observed across genders and school grades. In essence, sports participation exhibited an incremental positive relationship with resilience, suggesting that increased participation in sports leads to higher resilience. This finding is a novel contribution to the literature, and given its novelty, it is challenging to locate comparable evidence to either support or refute our findings. The association between sports participation and resilience in children and adolescents has never been looked at before in our research. Consequently, more studies, especially those utilizing longitudinal or experimental designs, should be conducted to determine whether or not this association can be replicated. If more robust evidence can be accumulated, the association between sports participation and resilience can be integrated into mental health clinical counseling, providing practical applications.

In summary, it is essential to recognize the inherent constraints of this research. First, we are unable to show a cause-and-effect association between the independent and outcome variables due to the cross-sectional design of our research. Secondly, due to the online survey nature of our study, all measures relied on self-reported questionnaires, introducing potential biases related to social desirability and recall. Thirdly, our study solely collected data on the frequency of sports participation, and this limited information might constrain a more comprehensive recognizing the association between children and adolescents’ sports participation and resilience. Fourthly, we have included as many control variables as possible, but there may still be some omitted control variables that could influence the results. Nevertheless, our study benefits from its large sample size and the adjustment for numerous controlling variables. Future research endeavors need to take care of the above identified shortcomings to provide more solid proof, ultimately allowing for the confirmation or negation of our findings.

## 5 Conclusion

This study contributes valuable evidence on the association between sports participation and resilience, shedding light on the potential of sports participation in promoting resilience among school-attending students. Notably, the positive role of sports participation appears consistent across genders and school grades. The observation of sports participation and resilience have a positive association implies that increasing students’ engagement in sports participation could be a strategic approach to bolster mental health. However, given the study’s limitations, further investigations with enhanced research designs, such as experimental studies, are warranted to validate and extend these research findings.

## Data availability statement

The original contributions presented in the study are included in the article/Supplementary material, further inquiries can be directed to the corresponding author.

## Ethics statement

The studies involving humans were approved by Shenzhen University’s Research Committee. The studies were conducted in accordance with the local legislation and institutional requirements. Written informed consent for participation in this study was provided by the participants’ legal guardians/next of kin. Written informed consent was obtained from the minor(s)' legal guardian/next of kin for the publication of any potentially identifiable images or data included in this article.

## Author contributions

XS: Formal analysis, Methodology, Supervision, Writing – original draft, Writing – review & editing. KXL: Data curation, Investigation, Supervision, Writing – review & editing. KL: Data curation, Investigation, Supervision, Writing – review & editing. XC: Data curation, Investigation, Supervision, Writing – review & editing. HF: Conceptualization, Methodology, Supervision, Validation, Writing – review & editing.
